# Decision Making on Telemedicine for Patients With Epilepsy During the Coronavirus Disease 2019 (COVID-19) Crisis

**DOI:** 10.3389/fneur.2020.00722

**Published:** 2020-06-26

**Authors:** Naoto Kuroda

**Affiliations:** Department of Pediatrics, Children's Hospital of Michigan, Wayne State University, Detroit, MI, United States

**Keywords:** epilepsy, COVID-19, telemedicine, decision making, algorithm, epilepsy surgery, novel coronavirus

## Introduction

Coronavirus disease 2019 (COVID-19) is a novel infectious disease caused by severe acute respiratory syndrome coronavirus 2 (SARS-CoV-2). The outbreak initially occurred in Wuhan, China, in late 2019, and is spreading globally (1). Society, in general, is required to adapt to the changes induced by the COVID-19 crisis.

Epilepsy is a neurological chronic disorder characterized by a spontaneous recurrence of unprovoked seizures. In the field of neurology, as well as other departments, telemedicine is recommended as an alternative option for outpatient practice during the COVID-19 crisis to avoid the risk of exposure to SARS-CoV2 (2). The clinicians are trying to change the practice style to telemedicine owing to the worldwide COVID-19 crisis. Telemedicine was already in use before the COVID-19 crisis for some patients who had difficulty accessing medical facilities. However, the COVID-19 crisis has made telemedicine an option that should be actively considered in most patients, not just some patients who have difficulty accessing medical facilities. In the field of epilepsy, the importance of telemedicine is emphasized during this crisis; however, no consensus about decision making is available (2).

**Figure 1:**
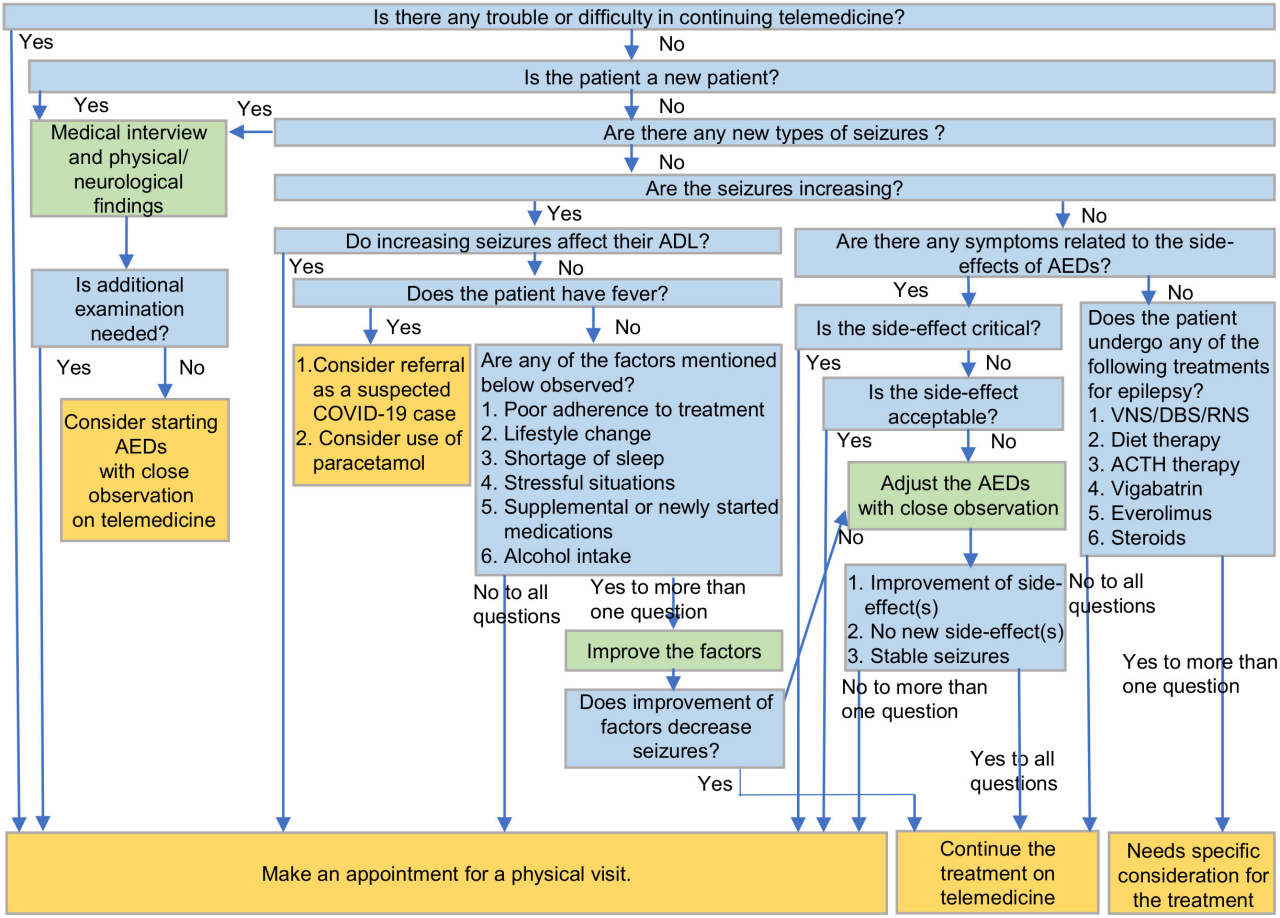
A decision-making tree to manage patients with epilepsy on telemedicine during COVID-19. COVID-19, coronavirus disease 2019; ADL, activities of daily living; AEDs, antiepileptic drugs; VNS, vagus nerve stimulation; DBS, deep brain stimulation; RNS, responsive neurostimulation; ACTH, adrenocorticotropic hormone.

The present study article emphasizes the decision-making process by clinicians for patients with epilepsy via telemedicine during the COVID-19 crisis. Considering the decision-making factors, a decision-making tree has been proposed (Figure 1).

## Decision Making on Telemedicine

### Strength and Weakness of Telemedicine

Telemedicine consultation between clinicians and patients with epilepsy has certain advantages. First, the patients can save time, consultation fees, and travel expenses required to visit their doctors (3). Second, Patients living in areas with poor medical resources can have access to professional doctors of the required fields (3). Third, patients can avoid public exposure, which is highly recommended during COVID-19 crisis (2).

In contrast, telemedicine has certain limitations compared to physical consultation. It is difficult to examine the patients online, the effectiveness is limited, and remains unauthenticated (4). In general, patients cannot undergo clinical tests associated with epilepsy such as blood test, cerebrospinal-fluid test, neuroimaging, and electroencephalogram, thereby indicating that patients requiring such examination have to physically visit the testing centers despite the exposure risk to SARS-CoV-2.

### Decision Making by Clinicians for Patients With Epilepsy via Telemedicine During COVID-19 Crisis

Based on the above-mentioned advantages and limitations of telemedicine, physicians should recommend both outpatient consultation at clinic and telemedicine appropriately. During telemedicine consultation, the doctors should decide whether the patients require a visit to the clinics.

#### Access to the System of Telemedicine

To continue telemedicine, the feasibility and accessibility for patients remain one of the most important factors. Some factors disturb the patients to continue the telemedicine (2). People who are blind or hard of hearing may be more inconvenienced by telemedicine. Some patients are unable to use a video-call system for a variety of reasons, such as difficulty accessing the internet, location, or financial situation. Patients in such situations should be encouraged to visit the clinics. It is more important to provide continued medical care.

#### New Patients

In the cases of new patients, we would collect general information, including clinical history, family history, seizure semiology, medical history, and birth and developmental history. In addition, we would check the physical and neurological findings. If additional testing is needed, including blood test, tap test, electroencephalogram, or neuroimaging, an appointment can be made for the patient to come into the clinic. In cases where more examinations are not needed, we can continue with telemedicine.

#### The Presence of New Seizure Type

The occurrence of seizure types would indicate acute symptomatic seizure due to onset of new diseases including COVID-19 (5). Even for patients with epilepsy as a neurological chronic disorder, the new types of seizures should be distinguished from the original seizures and must be examined in detail. The diseases which cause seizures include cerebrovascular disease, head trauma, central nervous system infection, auto immune encephalitis, and intoxication (6). We should also remember that psychogenic non-epileptic seizures are a disorder that is often difficult to differentiate from epilepsy (7). Therefore, epileptologists should confirm the presence of seizure types, differing from the original seizures.

#### Seizure Frequency

While consulting outpatients with epilepsy, an essential factor is the change in seizure frequency. Several factors affect the seizure control (8). If the seizure frequency increases, clinicians should consider all factors responsible for the loss of seizure control.

In the cases of increasing seizures, clinicians have to confirm several factors associated with seizure occurrence. Changing lifestyle, such as change of sleep duration, decreased medication compliance, and stressful/anxious situations can affect the seizure control. Alcohol consumption is a known trigger for seizures in patients with epilepsy. It is widely known that people under stressful situation tend to consume alcohol more frequently (9). Moreover, it is widely known that anxiety and mental stress can increase seizures (10). To control seizure, medication compliance is essential. During the COVID-19 crisis, lifestyle changes are bound to occur, which may decrease the medication compliance (11). Considering prevention or supportive therapy of COVID-19, patients with epilepsy might be exposed to various supplements or newly started medications. As reported, some antiviral therapy used for COVID-19 may interact with the antiepileptic drugs (AEDs) (12). According to The Italian League Against Epilepsy (https://www.lice.it/pdf/Antiepileptic_drugs_interactions_in_COVID-19.pdf), some AEDs (gabapentin, levetiracetam, lorazepam, pregabalin, clonazepam, ethosuximide, et al.) have relatively few interactions with antiviral medications and are easy to combine with them. O the other hand, some AEDs (carbamazepine, phenytoin, phenobarbital, et al.) have strong interaction with antiviral drugs. In addition, some combinations can induce fatal arrhythmias in patients. Therefore, clinicians should consider the effects of interaction between such medications and AEDs.

We should first confirm such factors, including medication adherence, lifestyle, shortage of sleep, stressful situations, alcohol intake, and supplemental or new medications. If there is no change in terms of these factors, we would consider about adjustment of AEDs. If the increase in seizures disturbs the patients' daily activities, physician could encourage making appointment for physical consultation, whatever the causes of exacerbation might be.

#### Patients' General State

Any person could be infected by SARS-CoV-2. Therefore, clinicians should ask the patients with epilepsy whether they have any symptoms of COVID-19. Similar to the general population, patients with epilepsy who have comorbidities, such as smoking, obesity, diabetes, heart disease, lung disease, and cancer, are at a higher risk of developing COVID-19 (13). Among the COVID-19 symptoms, fever should be appropriately monitored as it could decrease the threshold of seizures (14). If required, the clinicians should consider prescription of antifebrile medications. In particular, for patients with Dravet syndrome, the management of fever is critical (15). Moreover, it is reasonable to use paracetamol if patients with Dravet syndrome have a fever (16). In addition to patients with Dravet syndrome, those with epilepsy can also be prescribed antifebrile medications, such as paracetamol.

#### Side-Effect of AEDs

If the patients with increased seizure lack the aforementioned factors (medication adherence, lifestyle, shortage of sleep, stressful situations, alcohol intake, supplemental or new medication, and patients' general state) or when improvement in such factors does not decrease the seizure frequency, clinicians may consider adjusting the AEDs.

While adjusting AEDs on telemedicine, the side-effect of AEDs should be considered. Telemedicine allows us to weigh the side-effects of the treatment (17). In case of critical side-effects, clinicians may consider physical consultation. We may consider continuing the AEDs if the side-effects are mild and acceptable to both clinician and patients. However, if the side-effects are critical, an appointment at the clinic will be needed. We need to consider discontinuing or changing the therapeutic drug on telemedicine if the side effects are not critical, and are unacceptable to either the physician or the patient. If the side-effects do not improve despite decreasing or stopping the AEDs, the patient should be encouraged to physically visit the clinician for further evaluation.

#### Other Treatments for Epilepsy

Some patients with epilepsy resort to the diet-therapy to control seizures. Diet-therapy would be vulnerable under the effect of COVID-19 crisis owing to the difficulty of obtaining the required foods for diet-therapy (18). Therefore, the clinicians should confirm the compliance to diet-therapy on telemedicine. Few patients with diet-therapy would need online-dietary-counseling by dieticians.

Patients with epilepsy who underwent surgeries, such as vagus nerve stimulation, deep brain stimulation, or responsive neurostimulation, require the adjustment of parameter for stimulation. In the present scenario, clinicians have no alternative options to manage and control such procedure on telemedicine. In particular, patients who are not very long post-operatively or who need to have parameters set may need an actual visit even in a COVID-19 situation.

Some patients take medications, such as everolimus or corticosteroid, which affect the immune system. Under normal conditions, such medications are normally prescribed to control seizures (12). However, these medications may need to be discontinued for a short period for patients with exposure to SARS-CoV-2 or diagnosed as COVID-19 positive (19).

Vigabatrin is an AED often used for patients with tuberous sclerosis complex. Clinicians can continue prescribing this medication on telemedicine if approved by the opthalmologists, as it is known to cause visual field defect (20).

Some patients with infantile spasm might have undergone adrenocorticotropic hormone (ACTH) therapy for epilepsy (21). This therapy is generally provided on admission; however, during the COVID-19 crisis, physicians could consider using oral steroid as an alternative option for ACTH therapy on telemedicine to reduce the risk of virus exposure (21, 22).

## Conclusion

The present study summarizes and suggests the management of patients with epilepsy on telemedicine during the COVID-19 crisis. Epileptologists are required to manage their outpatients using telemedicine due to COVID-19 pandemic. Decision-making tree prescribed in this article would be helpful for the epileptologists to manage their patients on telemedicine. In COVID-19 situations, patients are exposed to various aspects of psychological stress. Therefore, in addition to decision-making, understanding and listening to the patient's stress in the telemedicine is important during the COVID-19 crisis. It is also important to establish a medical system that allows for the continuation of telemedicine in the long term, even after the COVID-19 crisis. Research will also be needed to evaluate the effectiveness and safety of telemedicine in the COVID-19 crisis.

## Author Contributions

NK: study/article design, data collection, and manuscript writing.

## Conflict of Interest

The author declares that the research was conducted in the absence of any commercial or financial relationships that could be construed as a potential conflict of interest.
